# A connection from stratospheric ozone to El Niño-Southern Oscillation

**DOI:** 10.1038/s41598-017-05111-8

**Published:** 2017-07-17

**Authors:** Desmond Manatsa, Geoffrey Mukwada

**Affiliations:** 1 Geography Department, Bindura University of Science, Bindura, Zimbabwe; 2 Earth System Physics, International Centre for Theoretical Physics, Trieste, Italy; 3 Geography Department & Afromontane Research Unit, Free State University, Qwa Qwa, SA 9301 South Africa

## Abstract

We use reanalysis and observational data to link the lower stratospheric ozone regulation of the ultraviolet radiation (UV-B) component of solar energy to ENSO modulation. Results indicate that during ENSO extremes, the Walker Circulation (WC) and Brewer Dobson Circulation are related to lower stratospheric ozone alterations east of the date line over the Pacific. These in turn are linked to upper tropospheric anomalous dipole temperature patterns on either side of the equator. The ensuing changes in geopotential height values do not only drive equatorial zonal wind anomalies in the upper troposphere that are reversed at the equatorial surface, but also impact on the intensity of the South Pacific High circulation. When the WC is enhanced, a La Nina type of circulation is indentified but if the circulation cell is inverted, the anomalous circulation results in an El Nino. Though the anomalous lower stratospheric ozone peaks during austral summer it is significant throughout the ENSO lifecycle. Hence, ENSO structure and variability are mainly linked to the lower stratospheric ozone instigated internal dynamics of the Pacific atmosphere. The ENSO forcing most likely originates from the ozone related regulation of the incoming solar UV-B radiation rather than the Pacific Ocean surface.

## Introduction

El Niño Southern Oscillation (ENSO) is a tropical Pacific phenomenon that is characterised by coupled oceanic (El Niño) and atmospheric (Southern Oscillation) components^1^ which in turn regulates the lower stratospheric ozone^2, 3^. Of late it was believed that stratospheric processes respond to but do not impact on the tropospheric flow. But recent observations and model results generated in the context of the annular modes indicate mutual coupling. Any slight perturbation to the lower stratospheric ozone is seen to induce large and robust anomalies in tropospheric circulation and temperature^4^ which are sufficiently strong enough to impact the surface^5, 6^. Although it is well known that a change in the Walker Circulation (WC) plays a pivotal role in the ENSO phenomenon, the source of the shift in the Pacific convective activity is still a mystery. However, what has been observed is that it is the upper-level westerly winds and not the surface zonal winds of the equatorial region which initially become anomalous^7^. In this regard, ozone related lower stratospheric circulation modulation over the Pacific could provide a possible explanation to ENSO development and modification.

The mechanism which causes and sustains ENSO is not fully understood whilst theories to explain ENSO development and sustenance^8, 9^ were put forward when the lower stratospheric ozone anomalies and surface circulation conditions were largely considered decoupled. The tropospheric WC variability is strongly coupled to ENSO. At the same time the Brewer Dobson Circulation (BDC)’s tropical upwelling system is a stratospheric phenomenon which predominantly involves the lower tropical stratospheric vertical ozone transport^10^. The BDC is composed of deep and shallow branches with the latter transporting ozone to the subtropics and mid latitude^11^. As such the extratropical ozone becomes inversely related to ozone amounts within the tropics^10^. At the same time, the coinciding peak transport period of the BDC with ENSO induced tropospheric convection east of the date line during NH winter, enables the WC’s zonal air transport to be linked to the lower stratospheric meridional air transport^12^. Consequently, ENSO related cross-tropopause ozone transport^13, 14^ should not only create ozone anomaly patterns in the tropical Pacific^15^ but dipole anomalies located on either side of the equator east of the dateline.

For decades, the spectral amount of solar radiation reaching the earth has been known to peak in the visible spectrum^16^. But, recently ref. 17 used the Plank’s postulate to show that the solar energy is much higher in the ultraviolet (UV) radiation spectrum whose wavelengths are less than those of the visible spectrum. Of this UV radiation, the most energetic UV-C is used up before reaching the troposphere where it is considered principally responsible for increasing the temperature by ~50 °C from the tropopause to the stratopause. Although the least energetic UV-A reaches the surface, it is generally not significantly perturbed. Ozone, which is in abundance in the lower stratosphere, predominantly uses the UV-B solar spectrum to warm the lower stratosphere during the Chapman’s Cycle^18^. Hence, the ENSO related changes in the ozone layer thickness should be linked to the proportion of the UV-B radiation that is absorbed in the lower stratosphere and that which penetrates to warm both the troposphere and the surface. This could explain why the ozone modulation has been known to be directly related to the lower stratospheric temperature while inversely related to the tropospheric and surface temperature^19^. It then follows that the solar energy changes through the UV-B due to the ENSO-related lower stratospheric ozone modulation^20^ should be able to account for the widespread changes in the Pacific tropospheric circulation and surface climate. Here we present evidence that suggests that the lower stratospheric ozone regulation has a verifiable impact on the surface climate of the tropical Pacific that is related to ENSO’s sustenance and probably development.

## Results

### Tropical Pacific contrasting troposphere and surface spatial temperature patterns

**Figure 1:**
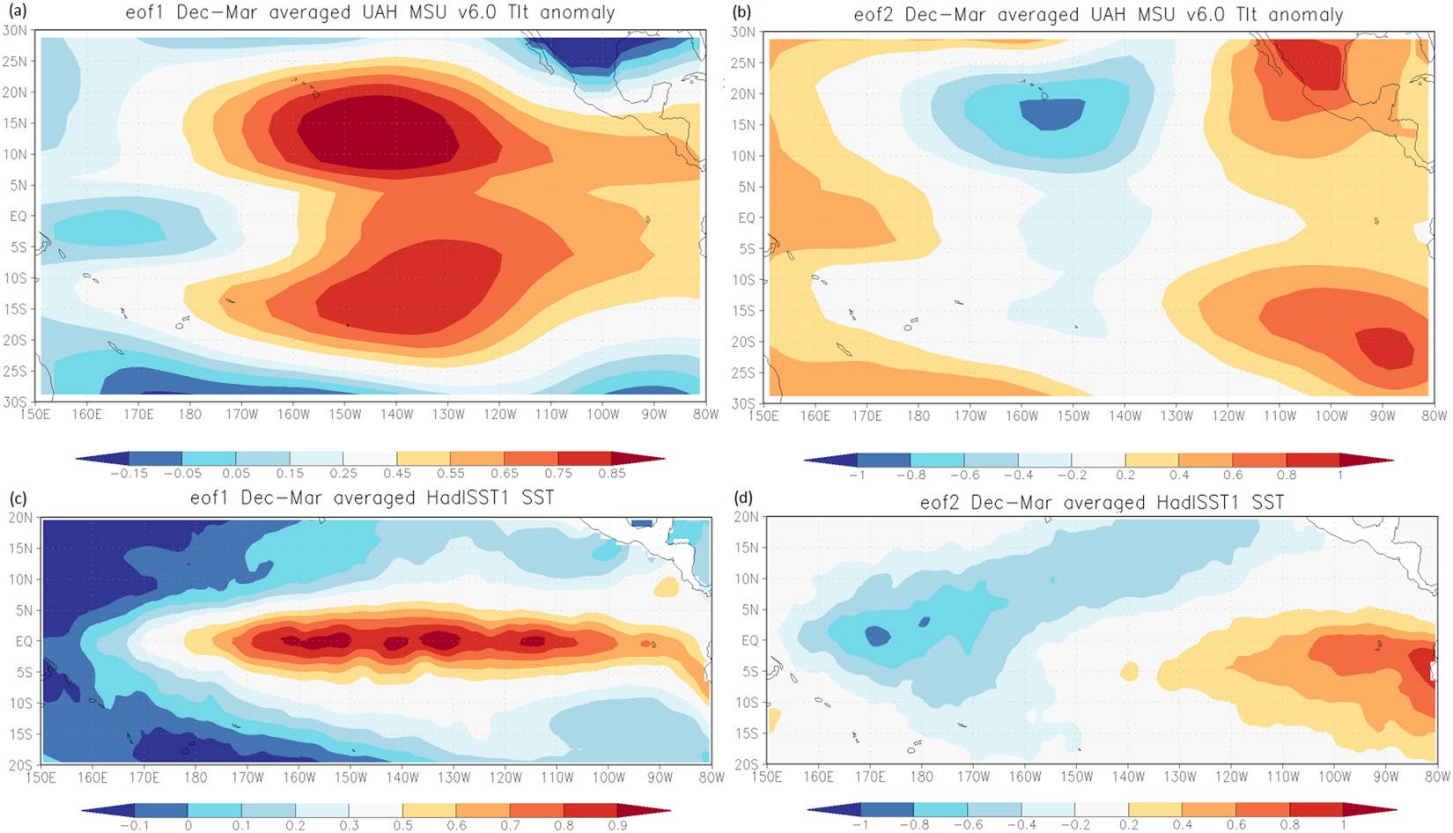
The respective spatial structure for EOF1 and EOF2 for the lower tropospheric temperature (**a**) and (**b**), and for the SST (**c**) and (**d**). The period is from 1979 to 2016 for the averaged months of December to March and the anomalies are with respect to 1981 to 2010. The EOF figure panels were generated using the online software from http://climexp.knmi.nl/eofform.cgi? UAH MSU v6.0 dataset is from http://www.atmos.uah.edu/data/msu/ and HadISST1 dataset is from http://hadobs.metoffice.gov.uk/hadisst/. However, caution in the interpretation should be taken as the HadISST1 dataset could be limited if utilized either for high resolution or spatial gradients and a minor discontinuity at the dateline can affect estimates of the variability in the region.

The primary focus of examining ENSO extremes is traditionally on the Pacific Ocean surface, where the equatorial SST anomalies and variations in sea level pressure over the eastern and western tropical parts of the ocean have been used to define the intensity of the events. Considering the atmosphere as a mere response to surface conditions has probably diverted attention from the important role played by the atmospheric component in previous ENSO analyses. To reflect on this bias, in Table 1a and b we show the explained variances in percentages of the dominant eigenvalues for the Pacific lower tropospheric air temperature and SSTs respectively, which have been truncated to 4 using a scree plot technique (not shown). The percentage of the variance explained by the dominant pattern for the former explains about 74% whilst that of the latter is about 71%. The second dominant pattern explains only 6% and 13% respectively, and was assessed to be significantly different from their corresponding first dominant pattern using ref. 21 criterion. Figure 1 represents the respective spatial loading patterns of the first two leading modes for tropospheric temperature and SSTs respectively. In Fig. 1a, the region with the highest loading amplitudes to the east of the dateline coincides with the region of maximum modulation where the vertical motion due to the WC interferes with that of the BDC during ENSO extremes. The first leading SST pattern depicted in Fig. 1c is referred to as canonical ENSO and the second EOF (Fig. 1d) which is characterized by an east–west dipole is known as ENSO “Modoki,”^22^. Interestingly, both ENSO flavors appear not to have easily identifiable features to relate them to any of the leading modes in the tropospheric temperature of the Pacific shown in Fig. 1a and b. This indicates that the processes directly responsible for the tropospheric air temperature variations could be different from those generating the surface temperature variation in the tropical Pacific. However, since the cross correlations between the two leading PC1s and Nino 3.4 have values well in excess of 0.93 (p ≪ 0.0001), this strongly suggests that the variability probably originates from the same source.

**Table 1:** Explained variances of the eigevalues for (a) the lower tropospheric temperature and (b) the Pacific SSTs presented in the respective regions of Fig. 1.

a				b			
#	eigenvalue	explained variance	cumulative	#	eigenvalue	explained variance	cumulative
1	1962.7	73.98%	73.98%	1	1299.3	71.38%	71.38%
2	159.29	6.00%	79.99%	2	235.92	12.96%	84.34%
3	112.43	4.24%	84.23%	3	66.326	3.64%	87.98%
4	90.676	3.42%	87.64%	4	60.631	3.33%	91.31%

**Figure 2:**
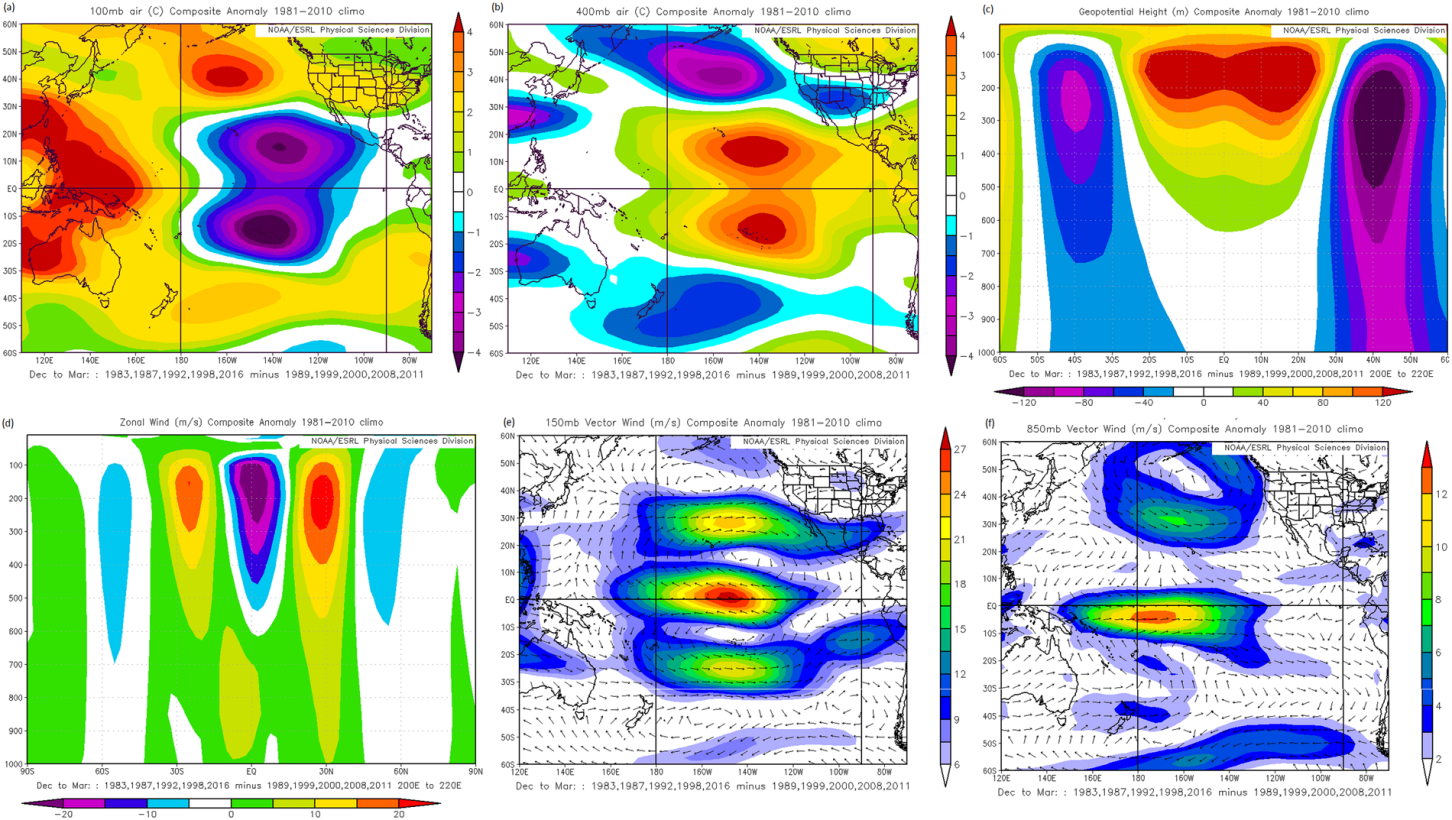
The El Nino minus La Nina mean difference composite spatial pattern for the; (**a**) 100 mb and (**b**) 400 mb level air temperature, 200°E–220°E averaged (**c**) vertical Z and (**d**) zonal wind slice, zonal wind at the (**e**) 150 mb and (**f**) 850 mb levels. The years comprising the composites are indicated in the inserts. The figure panels were generated using the online software from https://www.esrl.noaa.gov/psd/cgi-bin/data/composites/printpage.pl and the corresponding NCEP/NCAR^32^ datasets selected for construction of the composites are indicated in the inserts.

Figure 2 shows the ENSO composites in the vertical and horizontal spatial scales from the 100 mb level down to the near surface (850 mb level). The analysis of the 100 mb level is essential because variations near the tropopause are highly sensitive to lower stratospheric ozone changes and temperature^10^. This enables the detection of the impacts of extreme ozone anomalies in both the lower stratosphere and the adjacent troposphere by inferring both the zonal and meridional ozone transport over the Pacific due to the WC and shallow branches of the BDC respectively. In this regard, the inverse relationship in temperature that is prominent in Fig. 2 is probably due to the respective rising motion which depletes ozone in the tropics east of the dateline and accumulates it to the tropical Pacific west and mid-latitudes as a result of descending arms of the respective circulation cells.

It is interesting to note that the spatial Pacific temperature patterns near the lower stratosphere (100 mb level) are essentially reversed in the middle troposphere represented by the 400 mb level (Fig. 2a and b). Although this pattern is depicted as the dominant mode of the tropical temperature variability in the troposphere in Fig. 1a, it is not in any way replicated at the surface. Hence the latitude–longitude plots presented in Fig. 2a and b are insufficient as it is difficult to reconcile the tropospheric and surface temperature signatures which manifest considerably different structures. We therefore present the vertical structure (Fig. 2c) which simultaneously reveals the full complexity of the tropospheric and surface ozone related signatures through temperature related impacts on the geopotential heights (Z). This figure shows that the Z anomalies are more pronounced in the upper troposphere which indicates the location of the center of tropospheric response to the ozone related temperature changes. Hence it implies that the tropospheric response to the temperature anomalies does not originate from the surface but is a product of processes linking the lower stratosphere to the upper troposphere. However within the central tropical Pacific region, where the Z anomalies appear to be at their maximum, the corresponding significant changes are restricted to the middle troposphere and do not reach the surface. These changes in the tropical Pacific tropospheric Z anomalies are indicative of a strengthening of the zonal flow. This is because the resulting bi-Z vortices on either side of the equator provide a close representation of the north–south gradient in Z along the equator and thus the strength of the equatorial zonal flow depicted in Fig. 2d. These Z changes in the central tropical Pacific troposphere are accompanied by poleward and westward inverse changes that form an anomalous dipole in Z configuration on both hemispheres. The extratropical anomalies cascade downward to the surface of the subtropics in the SH but further north to the mid latitudes in the NH. As such in the SH, the Z changes occurring during austral summer period, are expected to considerably impact on the intensity of the South Pacific Anticyclone as it will be at its weakest strength during this period. The strong similarity between the Z pattern in this figure and the vertical correlation field of Z with the Nino 3.4 index (Fig not shown) reiterates the strong coupling between the Z changes and ENSO variability.

In Fig. 2e and f we illustrate the distinct circulation patterns which accompany the heating anomalies presented in Fig. 2a and b for the upper troposphere and near surface respectively. The presence of the two adjacent relatively strong anticyclones within the subtropics on either side of the equator in the central Pacific drives strong easterly anomalies along the equator. On the other hand the adjacent anticyclonic and cyclonic anomalies in each hemisphere steepen the pressure gradient between their respective vortices resulting in enhanced westerlies in the subtropics. Near the surface, the tropical circulation pattern seems to be detached from the middle and upper tropospheric circulation. But a fall in subtropical Z in the SH presented in Fig. 2c has resulted in the weakening of the South Pacific High hence the anomalous near basin-wide cyclonic circulation depicted in Fig. 2f. This in turn is linked to enhanced easterly anomalies along the equatorial region though with a bias towards the SH. In the mid-latitudes, the upper level anomalous cyclonic vorticity center in the upper troposphere appears to strongly translate to the surface below resulting in an equally strong cyclonic vorticity anomaly pattern. However, these changes seem not to imprint distinct circulation anomaly pattern on the North Pacific Anticyclone, and hence has minimal impact on the enhancement of the westerly equatorial surface flow.

The prominent upper-level Pacific vortices that developed from the ozone related rise (fall) in Z (Fig. 2c) due to the ensuing temperature changes should be responsible for the equatorial surface flow anomalies indicated in Fig. 2f. Hence, the zonal acceleration of the lower stratospheric equatorial flow due to ozone regulation is interesting in its own right due to its coupling to the Pacific equatorial surface. In the next section we show that the formation of the strong tropical Pacific High (Low) in the upper troposphere which cascades Z anomalies down to the middle troposphere is most probably due to the heating associated with the lower stratospheric ozone modulation.

### Link of ozone modulation to ENSO

**Figure 3:**
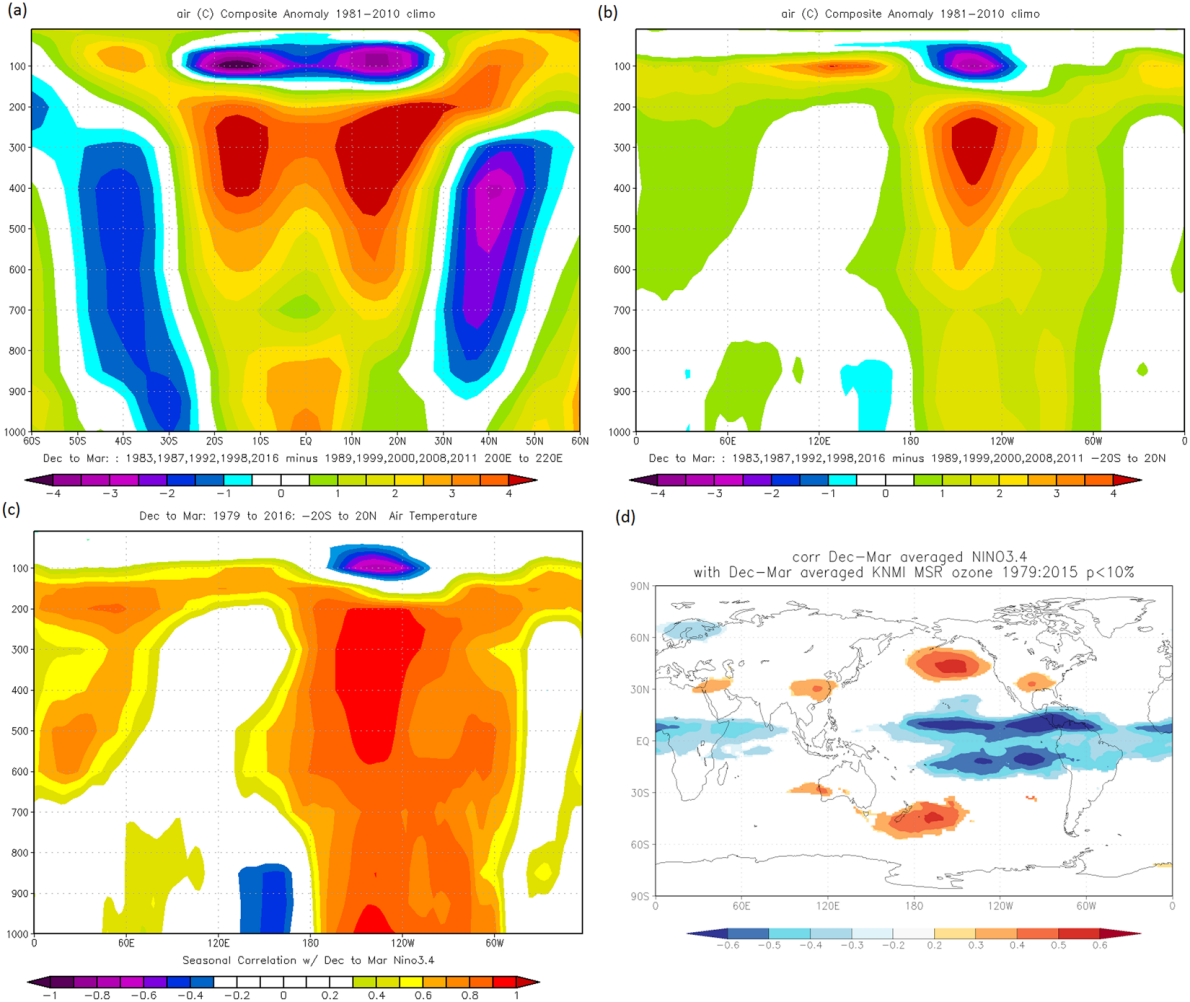
The El Nino minus La Nina mean difference composite spatial pattern for the (**a**) 200°E–220°E and (**b**) 20°N–20°S Z averaged vertical slice in temperature. Nino 3.4 correlation with (**c**) the 20°N–20°S temperature averaged vertical slice and (**d**) Total ozone column correlation field for the period 1980 to 2016. The years comprising the composites are indicated in (**a**) and (**b**). The figures (**a**) to (**c**) were generated from ref. 32 air temperature using the online software https://www.esrl.noaa.gov/psd/cgi-bin/data/composites/printpage.pl. Figure 3d was constructed from http://climexp.knmi.nl/corfield_obs.cgi with the ozone data are from http://www.temis.nl/protocols/o3field/o3mean_msr.php. Nino 3.4 data for the correlations are from https://www.ncdc.noaa.gov/data-access/marineocean-data/extended-reconstructed-sea-surface-temperature-ersst-v4.

The BDC enhances stratospheric tropical upwelling during El Niño phases and suppresses it during La Niña events^20, 23^. Hence, the upwelling modulation is able to adjust the low-ozone tropospheric air entering the lower stratosphere, which in turn influences lower stratospheric ozone concentration^20, 23^. The BDC has distinct upper and lower branches with the deep branch carrying the tropospheric air to the high latitudes while the shallow branch transports the air that dissents in the subtropics and mid latitudes^13, 24, 25^. Consequently, while enhanced tropical upwelling depletes the tropical lower stratosphere of ozone, the BDC accumulates ozone in the subtropics and high latitudes. As such ozone concentration in the tropics is inversely related to that on either side of the subtropics and high latitudes in the stratosphere^10^. This is depicted in Fig. 3d where the ENSO signature is evident in the tropics and mid-latitudes east of the date line as significant negative and positive correlations, respectively. However, tropospheric ozone concentrations are relatively high in its upper part mainly due to the downward ozone transport of lower stratospheric ozone which is achieved through the stratosphere-troposphere exchange process^13, 14^.

**Figure 4:**
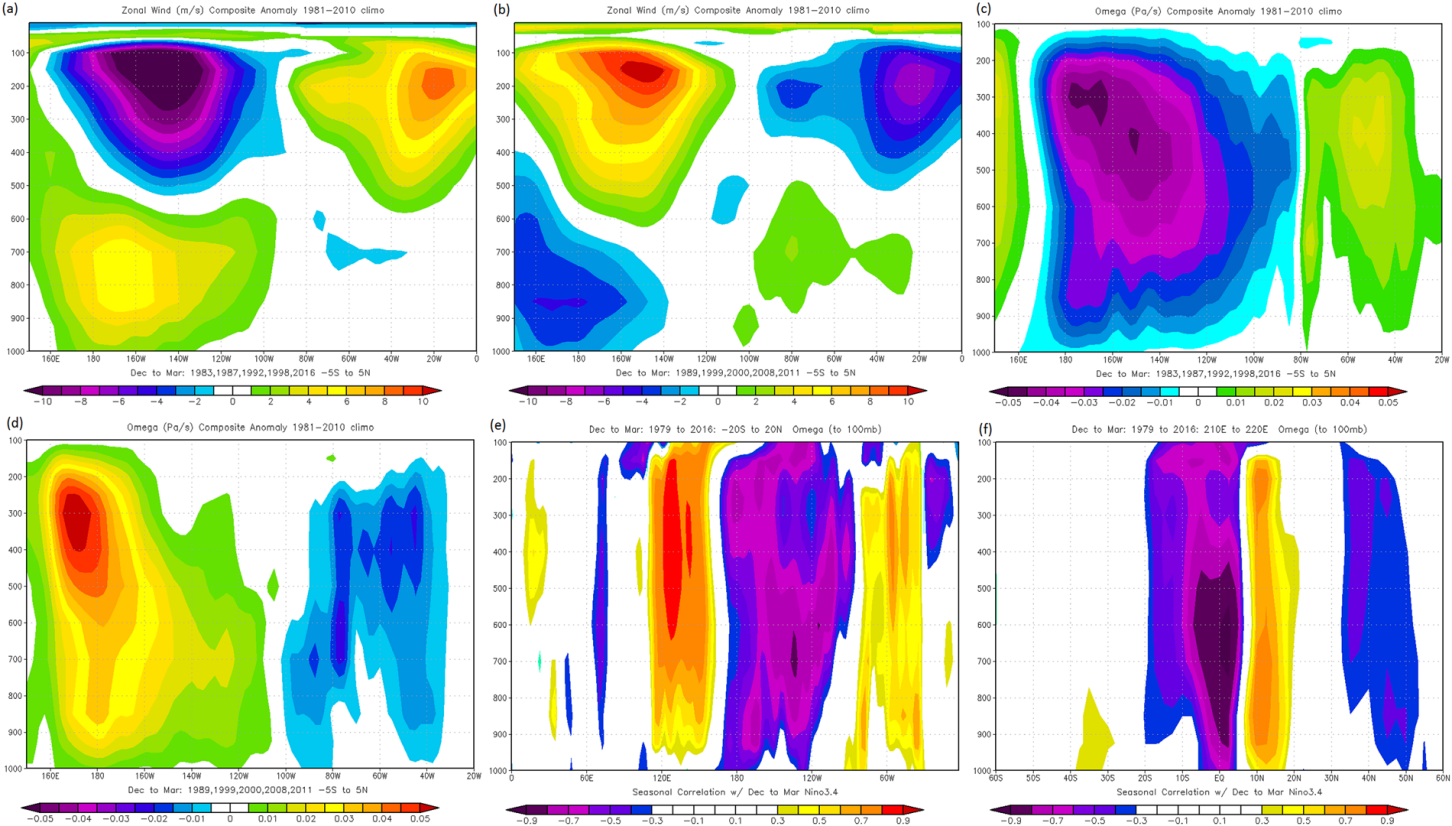
Spatial vertical composite pattern for the (**a**) El Nino (**b**) La Nina zonal wind events and, (**c**) El Nino (**d**) La Nina events omega values for the equatorial slice (5°N–5°S). The correlation values between Nino 3.4 and omega values along the (**e**) equatorial and (**f**) 200°E–220°E vertical slice. Nino 3.4 data for the correlations are from https://www.ncdc.noaa.gov/data-access/marineocean-data/extended-reconstructed-sea-surface-temperature-ersst-v4. Zonal and omega values are from NCEP/NCAR^32^.

Ozone principally absorbs incoming UV-B spectrum of solar radiation^17^, hence its depletion/enhancement leads to cooling/heating of the related atmospheric layer. In this respect we note in Fig. 3a that the enhanced lower stratospheric upwelling in a typical El Nino event which depletes the ozone, is associated with anomalously low temperatures in the lower stratosphere over the central tropical Pacific. This suggests that the excess UV-B which penetrates the thinner ozone layer, in turn is able to anomalously heat the ozone in the upper troposphere. The temperature anomalies centered over the upper troposphere are hardly associated with adiabatic rising (sinking) motions over the tropics, as Fig. 4f indicates negative omega anomaly values over the equatorial and tropical SH but positive anomalies over the NH tropics during the same El Nino event. At the same time anomalous deep convectional heating of the atmosphere does not produce such a pattern with maximum heating just below the tropopause. However the prominent subsidence in the tropical NH of this figure could be responsible for the enhanced anomalous heating observed in the upper troposphere relative to the corresponding region in the SH which is characterized by reduced warming due to anomalous uplift. In the subtropics, where the El Nino induced shallow BDC enhances the lower stratospheric ozone concentrations, the UV-B is able to anomalously heat the lower stratosphere at the expense of the tropospheric temperatures below. This suggests that the anomalous effect of the UV-B/ozone relationship over the tropical Pacific during ENSO is twofold; cooling (heating) the lower stratosphere and heating (cooling) mostly the upper troposphere during El Nino (La Nina). The corresponding reversed patterns in the subtropics and mid latitudes from the lower stratosphere to the upper troposphere are quite vivid in Fig. 3a. It should be noted that this observed dipole anomalous vertical temperature pattern in the atmosphere is not unique. Each year when the lower stratospheric ozone is enhanced in Polar Regions during local winter, less UV-B is observed to reach the troposphere and surface which causes regional cooling juxtaposed a warming of the lower stratosphere.

However, the relatively less cold lower stratospheric equatorial region (more ozone due to less upwelling between the two tropical branches of the BDC) that is conspicuous in Fig. 3a, implies that less UV-B penetrates to heat the equatorial upper troposphere, hence the cooler temperatures experienced there. It is also less likely that the UV-B is responsible for the anomalous heating observed at the surface over central Pacific equatorial strip as Fig. 1c illustrates that the SST variability is homogeneous from the dateline to the west of northern South America. In the lowermost atmosphere of Fig. 3a, the mean air temperature appears to be logically determined primarily by mean temperature of the mixed layer at the ocean’s surface. This suggests that the Pacific troposphere is predominantly anomalously heated from above and not from below. The implication is the prominent involvement of the lower stratospheric ozone regulation in anomalously heating most of the troposphere during ENSO extremes. Moreover, simulations forced with observed SST changes cannot fully explain the warming in the upper troposphere^26^, further strengthening the evidence that the warming does not originate from the ocean.

Since ENSO events are linked to coherent anomalies in the zonal ozone mean of the tropical lower stratosphere outside the Pacific^3, 27^ we note a corresponding anomalous heating of the upper tropical troposphere in that region during El Nino in Fig. 3b. The ENSO link of the tropical troposphere is also confirmed by the correlation pattern which depicts a similar configuration presented in Fig. 3c. Figure 3d implies that the significant impact of ENSO in austral summer on global ozone is predominantly through the combined impacts of the anomalous WC and BDC over the tropics and subtropics/mid latitudes of the Pacific, to the east of the date line. The anomalous action of the WC is zonal but more active to the east of the dateline where the reversal of its vertical branch occurs. The meridional BDC becomes anomalous when its poleward branches have their tropical upwelling enhanced through the additional influence of the anomalous vertical branch of the WC east of the date line. The anomalies are more pronounced in the NH where the BDC is accelerated more during austral summer than in the SH^28^.

The basic atmospheric dynamics dictate that the ozone related cooling/heating results in the strengthening/weakening of the Pacific stratospheric vortex. However, the underlying mechanisms coupling the lower stratospheric changes to variability in the tropospheric and surface flow in the form of ENSO remain unclear. This is despite the observation that the linkages between the ozone regulation and tropospheric circulation anomalies resembling ENSO are robust. Here we use the zonal wind and omega anomalies to represent the zonal atmospheric overturning circulation (the WC) above the tropical Pacific Ocean but focusing on the equatorial region to maximize the ENSO signal. Figure 4a and c show that during El Nino, the WC becomes inverted. Instead of the usually rising motion over the Maritime continent and northern South America, the upward flow is reduced resulting in an anomalous sinking branch with the anomalous rising branch west of the dateline. The equatorial upper flow becomes anomalously westerly with strong easterlies at the near surface (Fig. 4a). For the La Nina composites, the circulation is the reverse of the El Nino composites where the normal WC is essentially enhanced (Fig. 4b).

**Figure 5:**
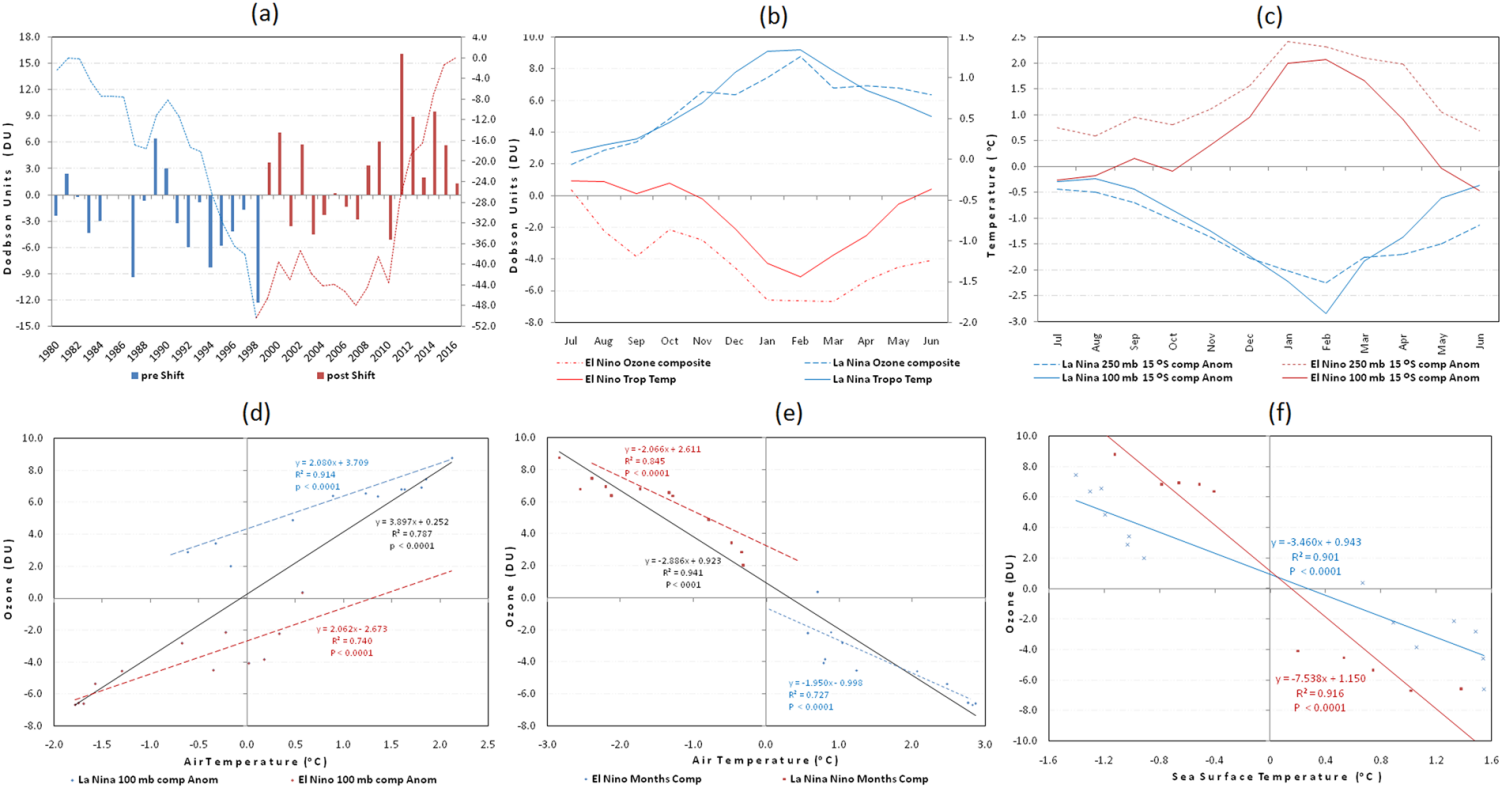
(**a**) Temporal manifestation of lower stratospheric ozone (bars) with the corresponding CUSUM time series superimposed (broken line). Monthly variation of the (**b**) ozone and (**c**) upper troposphere air temperature for El Nino and La Nina composites. Scatter plot for ozone against air temperature anomalies at the (**d**) 100 mb level and (**e**) 250 mb level for El Nino and La Nina composite months. (**f**) Scatter plot for zone against Nino 3.4 SST anomalies for the months August to February (blue) and March to July (brown). In the insert are the regression lines and their corresponding equations. The data is for the averaged boxed region (200°E to 220°E and 15°S to 15°N) for the period from 1980 to 2016. The ozone data are from http://www.temis.nl/protocols/o3field/o3mean_msr.php. Nino 3.4 data are from https://www.ncdc.noaa.gov/data-access/marineocean-data/extended-reconstructed-sea-surface-temperature-ersst-v4. The air temperature and SST data are from NCEP/NCAR^32^ and HadISST1 (http://hadobs.metoffice.gov.uk/hadisst/) respectively.

In this regard the alterations in the WC occur in conjunction with changes in temperatures in the lower stratosphere. Hence the fall in Z of the SH upper tropospheric subtropics (Fig. 2c) which is linked to the anomalous accumulation of the lower stratospheric ozone aloft, is translated to the surface where it weakens the equator ward zonal circulation of the South Pacific High (Fig. 2f). This in turn slows the ocean current that draws surface water away from the western coast of northern South America and reduces the upwelling of cold water from the deeper ocean. In the process, the thermocline is flattened, allowing warm surface water to build in the eastern part of the basin. The resulting altered anomalous zonal SST gradient across the western central tropical Pacific leads to the easterly trade winds converging across the equatorial Pacific to weaken, with the resultant westerly anomalies over the western central Pacific driving an El-Niño type event. Although we restricted our analysis to the equatorial region so as to maximize the WC signal, in the rest of the tropics, the enhanced upper and surface zonal wind anomalies are consistent with anomalously reduced strength of the equatorial WC typical of the El Niño episodes. In the case of the ENSO cool phase, the changes in the wind field are driven by anomalous lower stratospheric heating which is coupled to strong equatorial westerly (easterly) wind anomalies in the upper troposphere (surface). This is consistent with the observed anomalous strengthening of the South Pacific High which enhances upwelling over the equatorial eastern Pacific leading to a La Nina type event. These changes appear to result from coupled ocean-atmosphere feedback in which, easterly winds cause the sea surface temperature to fall in the east, magnifying the zonal heat contrast and hence amplifying the initial cooling by the southerlies^27^.

**Figure 6:**
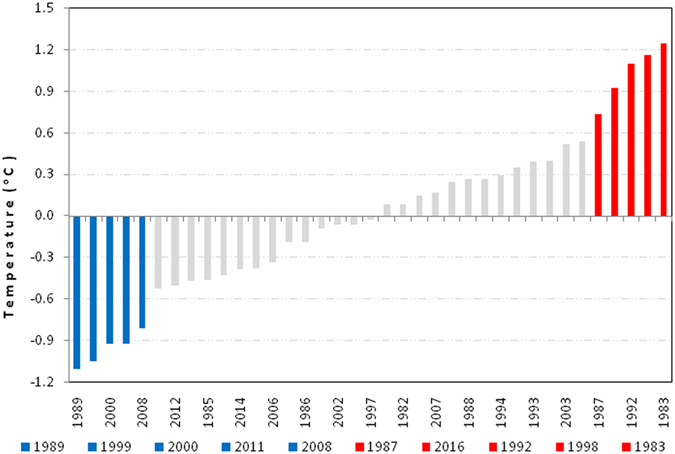
Annual variability of El Nino (red bar) and La Nina (blue bars) episodes based on the Nino 3.4 SST region averaged from December to March from 1979 to 2016. The ENSO extreme events have SST values exceeding ± 0.5 °C and are arranged in accenting order. The Nino 3.4 data were downloaded from https://www.ncdc.noaa.gov/data-access/marineocean-data/extended-reconstructed-sea-surface-temperature-ersst-v4.

The concurrent strengthening of the upper tropospheric zonal winds and the lower stratospheric ozone related temperature modulation makes it more likely that ENSO may owe its sustenance to internal tropospheric dynamics that are engineered from ozone regulation of the lower stratosphere. The observational evidence in Fig. 4a and b suggests that the lower stratospheric ozone depletion perturbs the upper tropospheric circulation in a manner that enhances the equatorial zonal wind anomalies. The vertical motion of the WC is represented by Fig. 4c and d. Here we observe that equatorial convection, which is represented by omega values is not symmetrical for El Nino (Fig. 4c) and La Nina events (Fig. 4d), where unlike in the former, convection for the latter is shifted more to the west of the dateline. Hence, while the Nino 3.4 region could be appropriate to determine El Nino events, it could be more accurate to characterize La Nina events using only the Nino 4 region where vertical motion anomalies are at their maximum to the west of the dateline. This observation is consistent with the National Oceanic and Atmospheric Administration (NOAA) recommendation^29^. This may explain why in Fig. 6, the ENSO SST anomalies are skewed toward warm events when the Nino 3.4 region is used to characterize both ENSO extremes.

Figure 4a to d implies that the significant anomalies in the circulation are more pronounced in the upper troposphere than near the surface. This puts into doubt the current understanding that the WC circulation is predominantly driven (sustained) by oceanic rather than atmospheric processes. Moreover, up to now a sound explanation is still lacking as to why the easterly surface winds slacken or even reverse resulting in the displacement of the warm waters from the western Pacific to the central and eastern Pacific Ocean basin. It could be that the anomalous ocean warming in the central and eastern Pacific as a response to lower stratospheric ozone depletion assists in shifting the rising branch of the WC to the east of 180°. It is at this location that the WC ascending branch reinforces the upwelling of the shallow branch of the BDC that descents in the mid-latitudes. In Fig. 4e we indicate that it is not the whole of the tropical WC which is significantly impacted on during ENSO but mostly the opposing branches over the eastern tropical Pacific and that over the Maritime continent and northern South America. Figure 4f further illustrates that the SH is negatively coupled with omega values though more intensely over the equatorial region whilst the NH part is positively correlated. This implies oppositely signed omega values within the tropics during a particular ENSO phase resulting in differential adiabatic heating which translated into asymmetrical modulation of the lower stratospheric ozone related heating within the tropics that is conspicuous in the upper troposphere of Fig. 3a.

### Relationship between Total-Column Ozone and ENSO lifecycle

Although in the earlier analysis we focused on the December to March period so as to enhance the WC and BDC signal on ENSO related variability, it is essential to show that the lower stratospheric ozone regulation is not sudden but gradual, impacting on the whole of the ENSO lifecycle. But before establishing this novel connection, we first illustrate the sustained annual linkage between ENSO and lower stratospheric ozone. The temporal manifestation of ozone in Fig. 5a shows a positive shift from 1998 which coincides with the shift to La Nina like events^30^. This is consistent with recent work which links La Nina events to lower stratospheric ozone surplus and deficit during El Nino events^20^. The observed increase in lower stratospheric ozone could explain the hiatus in warming rate in the tropospheric temperatures over the tropical Pacific from 1997, which could not be captured by coupled atmosphere-ocean general circulation models^31^.

By assuming the ENSO cycle to be essentially represented from July through to June the following year, we use the ENSO extremes determined from Fig. 6, to plot the monthly changes in lower stratosphere and upper troposphere for the ozone and air temperature composites. Figure 5b and c illustrate that the monthly evolution of both ozone and air temperature in the lower stratosphere and upper troposphere respectively, are highly inversely related, with peak values being attained from December to March for both El Nino and La Nina composites. This indicates that although the lower stratospheric ozone regulation and ENSO relationship is more prominent from December, the coupling between the two is evident throughout the ENSO lifecycle. The scatter plots for ozone against lower stratospheric air temperature and upper troposphere show that the relationships are not only uniquely highly coupled for El Nino and La Nina events at monthly time scales, but are on the overall also strongly related (Fig. 5d and e). However, as illustrated in Fig. 5f, the relationship between ozone and the Nino 3.4 SSTs appears not to be linear for all the 12 months. We note that the strong linear association can be divided into two distinct periods, from August to February and from March to June as a single regression line is no longer adequate to simultaneously represent the association for all the months. A closer analysis indicates that in the former months, ozone values are increasing (related to positive feedback) as opposed to the latter months when the ozone values are falling (related to negative feedback). This indicates that whilst ozone and the tropospheric temperatures are significantly linear, there is a different mechanism which relates the lower stratospheric ozone to the equatorial SSTs on monthly time scales.

## Conclusions

Tropical upwelling east of the date line through the coinciding impact of the WC and BDC creates centers of similar ozone related temperature anomalies within the tropics but which are out of phase with anomaly centers related to the shallow BDC subsiding branches in the subtropics. The resulting temperature related changes in Z values are linked to circulation vortices which generate equatorial zonal wind anomalies in the upper troposphere which are out of phase with those developed at the surface. Compensating vertical motion creates the anomalous WC cell within the tropics, which when reversed results in an El Nino type of surface circulation. In case of enhanced WC, a La Nina type of event develops. Therefore, the structure and variability of ENSO is mainly linked to the lower stratospheric ozone instigated internal dynamics of the tropical atmosphere which results in an expression of zonal wind variability along the Pacific equatorial region, both at the surface and upper troposphere. It is therefore more likely that the energy which drives ENSO does not originate from the surface but from the ozone related regulation of the incoming solar UV-B radiation.

We showed that the ozone regulation does not only peak during austral summer but is significant throughout the ENSO lifecycle. Therefore it can be argued that ENSO initiation is primarily a result of a slight central Pacific tropical perturbation in atmospheric physical and chemical properties that determine the lower stratospheric ozone equilibrium and how much of the solar UV-B energy anomalously warms the upper troposphere. This to some extent perturbs the WC which is amplified through ozone regulated process that grows through positive feedback mechanisms until the ENSO peak is reached during austral summer before progressively dying out during autumn. In this way the lower stratosphere could provide an “external” forcing on the tropical Pacific upper troposphere on intra-seasonal timescales that vary interannually and manifesting as ENSO extremes. Feedback mechanisms among the lower stratosphere, upper troposphere and surface may explain why once initiated, the ENSO event mostly completes its normal lifecycle. However it has to be acknowledged that our confidence in the linkages between the ozone regulation and ENSO which we have presented here is not exhaustive, primarily due to the lack of a quantitative and prognostic theory for dynamical coupling between the stratospheric and tropospheric circulations. But if the observations in this work are supported through modeling experiments, then the ozone/UV-B forcing can become the primary driver of solar induced tropical tropospheric climate variability.

## Data and Methods

To analyse the atmospheric fields we used the monthly National Centers for Environmental Prediction-National Center for Atmospheric Research (NCEP-NCAR) reanalysis on a 2.5° × 2.5° grid^32^. For the sea surface temperature anomalies (SST), the Hadley Centre Global Sea Ice and Sea Surface Temperature (HadISST) and the Extended Reconstructed Sea Surface Temperature version 4 (ERSST.v4) were used. The Micowave Sounding Units (MSU) measured lower-middle tropospheric temperature Spenser and Christy^33^ to represent the average tropospheric temperature. The ozone layer measurements as estimated by total ozone column data were derived from KNMI multi-sensor re-analysis^34^.

The data were selected for the period 1980–2016 so as to cover the post satellite period. The monthly anomalies were computed by removing the climatological (1981–2010) monthly means so as to conform with the current World Meteorological Organization recommendation. To identify El Niño and La Niña events in the tropical Pacific the Nino 3.4 region is used because it includes the western half of the equatorial cold tongue region. As such the region provides an added measure of essential alterations in SST gradients which could have resulted from displacements of deep tropical convection and atmospheric circulation. To capture mostly the mature ENSO extreme events which provide higher signals, we extracted only those anomalies that exceed the threshold of ±0.5 °C, for the averaged period from December to March. This criterion indentifies 5 events each of El Nino and La Nina phases which are presented in Fig. 6. The distribution of ENSO SST anomalies in this figure is skewed toward warm events because for consistency we used SST values in the Niño 3.4 region to also determine La Niña episodes. Otherwise it is more preferable to identify the cold events using the Nino 4 region as the region is normally characterized by SSTs at or above the 28 °C threshold for deep convection throughout the year^29^.

The Empirical Orthogonal Function (EOF) also known as the Principal Component Analysis (PCA) was used to examine the leading patterns and temporal variations of lower tropospheric Pacific air temperature variability. Unlike a single boxed region which is unable to distinguish between multiple large-scale patterns of variability, the EOF is able to isolate independent and co-varying patterns across a large spatial domain. The significance of the PCs was evaluated using ref. 21 criterion for identifying well-separated climate patterns. Though the ordering of the PCA is sensitive to the spatial domain considered, the two leading tropical Pacific SST patterns which we identified from the domain of 30°S–30°N, 130°E–80°W, also appear in a PCA of global SST and tropospheric air temperature anomalies.

In this work CUSUM is a sequential analysis technique used for shift detection in data by calculating the cumulative sum of anormalies using the following formular ${{\rm{S}}}_{{\rm{m}}}=\sum _{i=1}^{m}({x}_{i}-\bar{x})$ where S_m_ is the sum for m years, *x*_*i*_ is the observation during the m^th^ year and $\bar{x}$ is the data mean. The significance of the linear trends was evaluated using a two-tailed Student’s t test. We analysed ENSO extreme characteristics based on a symmetric assumption through composites of ENSO events and spatial correlation maps that incorporate both phases of ENSO^35^. Normally the significance of composite anomalies is determined by conducting the mean differences test using a t test or various resampling/Monte Carlo approaches. But due to the complexities resulting from interpretability^36^ which renders these usual tests of significance invalid, we did not attempt any statistical analysis. Instead we relied on an approach that merely enhances interpretability.
